# Primaquine in vivax malaria: an update and review on management issues

**DOI:** 10.1186/1475-2875-10-351

**Published:** 2011-12-12

**Authors:** Deepika Fernando, Chaturaka Rodrigo, Senaka Rajapakse

**Affiliations:** 1 Department of Parasitology, Faculty of Medicine, University of Colombo, 25, Kynsey Road, Colombo 8, Sri Lanka; 2 Department of Clinical Medicine, Faculty of Medicine, Colombo, Sri Lanka

**Keywords:** Primaquine, *Plasmodium vivax*, Vivax Malaria, Prophylaxis, Radical cure

## Abstract

Primaquine was officially licensed as an anti-malarial drug by the FDA in 1952. It has remained the only FDA licensed drug capable of clearing the intra-hepatic schizonts and hypnozoites of *Plasmodium vivax*. This update and review focuses on five major aspects of primaquine use in treatment of vivax malaria, namely: a) evidence of efficacy of primaquine for its current indications; b) potential hazards of its widespread use, c) critical analysis of reported resistance against primaquine containing regimens; d) evidence for combining primaquine with artemisinins in areas of chloroquine resistance; and e) the potential for replacement of primaquine with newer drugs.

## Introduction

Primaquine, an 8-aminoquinoline, has been approved for treatment of malaria since 1952 by the Food and Drug Administration (FDA), United States [1]. Six decades after its official licensing, primaquine still holds a unique and unchallenged place in anti-malarial regimens of cure and prophylaxis [2]. It is the only drug proven to be effective, and licensed to eliminate, the hypnozoites of *Plasmodium vivax *and *Plasmodium ovale*. Though primaquine is effective, unique and irreplaceable, it is also associated with serious hazards and side effects, such as its ability to precipitate haemolysis in glucose-6-phosphate dehydrogenase (G6PD) deficient individuals [3]. This prohibits its use in key groups, such as pregnant women [4]. The current uses of primaquine in vivax malaria are threefold: for radical cure of patients with confirmed parasitaemia; for causal prophylaxis; and for terminal prophylaxis [1]. In addition, it is used as a gametocytocidal agent in falciparum malaria.

This update and review deals with the current role of primaquine in treatment of vivax malaria. It will explore this topic on several fronts namely: a) evidence of efficacy of primaquine for its current indications; b) potential hazards of its widespread use c) critical analysis of reported resistance against primaquine containing regimens d) evidence for combining primaquine with artemisinins in areas of chloroquine resistance and e) the potential for replacement of primaquine with newer drugs.

## Methods

A MEDLINE search was performed for all articles with the key word 'primaquine' and 'Vivax' in any field. The search was restricted to articles published in all languages within the last two decades (1991-2011). There were 480 abstracts in the original search with these restrictions. The software Endnote X3 (Thomson Reuters, Carlsbad, CA 92011, USA) was used to filter articles. Bibliographies of cited literature were also searched. All abstracts were read independently by the three authors, and key articles were identified based on a consensus among all authors. Ninety two articles (including 40 clinical trials and 14 reviews) were selected for the final synthesis based on the relevance to the topic. The search was restricted to the last two decades to avoid redundant data and to select more recent evidence. However, related or cited papers of crucial trials before this period have also been included. Other papers included were case reports, opinion papers, treatment guidelines, experimental laboratory studies and cross sectional analyses of endemic populations. The epidemiological data and guidelines for treatment were downloaded from the websites of international agencies, such as the World Health Organization (WHO).

### Primaquine: Indications and efficacy

Primaquine has a unique and powerful role in the prevention and cure of malaria. It is the only FDA licensed drug that can destroy all liver stages (hypnozoites and schizonts) of the parasite. The drug has been licensed for use since the 1950s, though its mechanism of action is not yet fully understood. It is thought to interfere with the cellular respiration of the parasite by generating oxygen free radicals and deregulating the electron transport [1].

Before detailing the trial evidence on the efficacy of primaquine for its different indications, we would like to emphasize several issues that need to be remembered when interpreting data. Primaquine is not administered in isolation. It is co-administered with a blood schizontocidal agent and the total effect is a reflection of the synergistic efficacy of the schizontocidal agent and primaquine. Similarly, reported efficacy (or lack of it) depends on many other confounding factors such as the geographical region in which the trials have been conducted (equatorial, oceania or temperate regions), local strains of *P. vivax*, drug compliance, appropriate dosing according to bodyweight, duration of follow up, inability to differentiate re-infection from a relapse (indicating treatment failure), and small numbers in trial arms. Furthermore, results gained from controlled trial conditions may not be applicable when the drug is administered to the population, especially with regard to compliance. In determining the side effect profile, many trials have filtered out G6PD deficient individuals through initial screening; this may not always be possible with mass drug administration. These issues are discussed in detail in the relevant sections below.

The traditional indications for primaquine in vivax malaria are threefold [1]: primary prophylaxis, terminal prophylaxis, and radical cure.

#### Primary prophylaxis

Since the parasite must successfully complete its hepatic stage before entering the erythrocytic stage, primaquine is the ideal agent to use as primary (causal) prophylaxis against *P. vivax *and *ovale *species. It is recommended that 0.5 mg base/kg/d of primaquine (maximum 30 mg per day) should be started one day before exposure and continued daily till seven days after the end of exposure [1]. Four randomized placebo-controlled trials in Asia and South America have established the efficacy of primaquine for this indication (i.e., primary prophylaxis against vivax malaria) to be over 85%. Baird et al. in a non-randomized open label trial in Irin Jaya, Indonesia assessed the prophylactic efficacy of 0.5 mg/kg/d of primaquine administered every other day against weekly chloroquine 5 mg base/kg for 16-19 weeks [5]. The protective efficacy of the primaquine arm for vivax malaria was over 90%. These findings were confirmed in another placebo-controlled double blind randomized study, which showed the protective efficacy of primaquine (for vivax malaria) to be 93% with a significantly lower infection rate compared to placebo [6]. A third placebo-controlled double blind study in the same locality showed similar prophylactic efficacy of primaquine against vivax malaria [7]. Soto et al. assessed the protective efficacy of primaquine 30 mg/d against a placebo in non-immune Colombian soldiers and found it to have 85% efficacy in preventing vivax malaria [8].

While all these trials demonstrate an impressive efficacy rate of causal prophylaxis, it must be noted that all were small-scale trials (largest number in a treatment arm was 126 patients). Three out of four trials have been conducted in the same geographical area in Indonesia, and show similar results of high efficacy. This obviously raises doubts of its application to other geographical areas which may have different parasitic strains. In fact, in the Colombian study, even when they accounted for symptomatic cases of malaria (not parasitaemia), the protective efficacy was only 85%. It is notable that the situation in Africa is totally unaccounted for except for a retrospective study of Israeli travellers to Ethiopia. In this study, Schwartz et al. report almost 100% protective efficacy of primaquine 30 mg/d prophylactic dosing to prevent vivax malaria, which was significantly better than that of doxycycline or mefloquine [9]. However, the protective efficacy in Africa is yet to be assessed in a prospective randomized controlled trial.

Limited trial data, hazards of side effects, and the cost effectiveness of administration (prevention of malaria vs. costs of assessing the G6PD enzyme status) make primaquine an undesirable agent for causal prophylaxis at the moment. It is not currently licensed for this indication. However, when recipients are intolerant of other prophylactic medications, primaquine can be used as an alternative in non-G6PD deficient individuals [1].

#### Terminal prophylaxis

Primaquine is suggested to have a role in terminal prophylaxis, i.e., medication taken towards the end of the exposure period. This is based on the hypothesis that, in vivax malaria, relapses occur due to liver hypnozoites and a dose of primaquine should be administered to clear this hepatic reservoir to avoid relapses. The currently recommended dose for this purpose is also 0.5 mg base/kg/d (maximum of 30 mg/d) for 14 days which should overlap with a blood schizontocidal agent [1]. Chloroquine and primaquine are usually used in combination for this purpose [10] on the premise that chloroquine enhances the hypnozoite clearance of primaquine. However, Soto et al. have demonstrated that the combination of primaquine and chloroquine did not add to the protective efficacy against falciparum malaria, or, more surprisingly, against vivax malaria (as *P. vivax *was sensitive to chloroquine in this locality) when compared with primaquine alone [8,11]. Though the trial was carried out in a limited number of (< 200) individuals in Colombia, this interesting aspect has not been examined in detail by anyone else. In our opinion, the role of primaquine in terminal prophylaxis (in adult, non-immune, non-pregnant travellers) needs to be re-assessed with regard to cost and benefit. Even if the costs of testing for G6PD status are included, it might prove cost-effective in preventing local outbreaks in non-immune communities which result from relapse infections occurring in travellers returning home from malaria endemic areas. However it is noted that it may be impossible to screen each and every traveler visiting a malarial area for G6PD status. The benefit of primaquine terminal prophylaxis can be improved if it is administered to individuals with an intense and prolonged exposure to vivax malaria. Currently there are no guidelines on this.

#### Radical cure

The third indication for primaquine is that of radical cure [1]. Primaquine at a dose of 0.5 mg base/kg/d in two divided daily doses (maximum of 30 mg/d) for 14 days is a better recommendation to clear the liver hypnozoites when given together with a blood schizontocidal agent. Shorter courses of primaquine (5 days therapy) and lower doses (15 mg/d) have been shown to be ineffective in preventing relapses [12-17]. These studies were conducted in India, Sri Lanka, the Brazilian Amazon, Colombia and Afghan refugee camps in Pakistan, and had follow up periods from three months to one year. Until recently, the recommended dose for radical cure with primaquine was 15 mg/d for 14 days (in combination with chloroquine). This dose was agreed upon after observing the historical data of US soldiers returning from the Korean War and also taking in to account that primaquine at this dose was less likely to precipitate a haemolytic crisis with G6PD deficiency [1]. However experimental studies with different strains of *Plasmodium *have shown that the dose of 15 mg/d is ineffective in preventing relapses [1]. Furthermore, there have been multiple reports of treatment failure from various parts of the world where the older regimen of primaquine had been administered, although some of these may be due to poor compliance (data from Korean peninsula, Afghanistan, India, Vietnam, Thailand) [18-21]. Still, it had been shown that for individuals with a higher bodyweight (> 70 kg), a 15 mg/d primaquine dose was ineffective in preventing relapses even with good compliance [1,22]. Therefore the current recommendation of the Center for Disease Control (CDC), USA for use of primaquine for radical cure stands at 0.5 mg/kg/d for 14 days (maximum of 30 mg/d) [1]. Nonetheless, the World Health Organization recommends a dose of 0.25 mg/kg/d for 14 days except in Oceania and Southeast Asia [23]. Though the recommendation is such, the WHO acknowledges that primaquine in doses from 0.25 to 0.75 mg/kg/d are effective for radical cure when given for 14 days. The reason for recommending the lower dose may be the concern about possible precipitation of G6PD deficiency with higher doses. Still, with the popularization with cheaper methods for screening of G6PD deficiency (see below) and with reported cases of treatment failure with the lower doses (0.25 mg/kg/d), we suggest the 0.5 mg/kg/d dosing to be more appropriate for the radical cure of uncomplicated vivax malaria.

Finally, primaquine can be used to prevent the transmission of falciparum malaria. In patients treated with chloroquine or artesunate, terminal dosing with primaquine is effective in eradicating circulating gametocytes, which are infective to the vector/s [24]. Thus, it acts as a chemoprophylactic agent at the community level by interrupting the transmission of falciparum malaria. Interestingly, this has an impact on preventing cases of vivax malaria as well. The appearance of vivax infections following uncomplicated falciparum infections has been observed and reported by several authors [25-27]. The occurrence has been frequently noted in analysis of several patient cohorts of malaria trials, and thus cannot be considered a random phenomenon. Lin et al. have recently showed (in Cambodia) that falciparum gametocytaemia at the time of presentation of the febrile illness in uncomplicated falciparum infections is a marker of subsequent short-term infection with *P. vivax *[25]. The authors propose two theories in this regard. One is that in areas where both infections predominate, infection with one parasite means exposure to other as well. Therefore, a patient harbouring falciparum infection (manifested) may also have hypnozoites from a previous vivax infection as well (dormant). As the falciparum infection clears, the vivax gets activated. Secondly, it is possible that co- infection with a second parasite species (*P. vivax*) pushes *P. falciparum *to provide more gametocytes (shift of the equilibrium from asexual to sexual forms). Either way this confirms the place of primaquine in treatment regimens for falciparum infections. However, the standard 45 mg single dose given for gametocyte clearance is not effective in preventing subsequent vivax infections [26]. Some authors therefore recommend that a full course of primaquine should be initiated after a risk stratification process (to minimize risks and hazards of primaquine side effects) to patients with falciparum infections living in areas where both *P. falciparum *and *P. vivax *are found [25].

### Barriers to mass prescription: How safe is primaquine?

The most feared complication of primaquine administration is the precipitation of haemolysis in G6PD deficient individuals. Primaquine may also cause clinically non-significant haemolysis in non-G6PD deficient individuals as well. Other major side effects include; methaemoglobinaemia, hypersensitivity reactions and gastrointestinal disturbances [1]. In individuals without G6PD deficiency, trial evidence indicates that primaquine is well tolerated in therapeutic doses with minimum side effects. However, it is currently contraindicated in G6PD deficiency, NADH methaemoglobin reductase deficiency, known hypersensitivity to primaquine and pregnancy as recommended by the Center for Disease Control [1]. The recommendation by the British Medical Association is also similar [28]. It is considered safe for use in breastfeeding mothers as long as the G6PD status of the infant is tested and known. It is not recommended for use in children under 4 years of age.

Though these contraindications exist on paper, in practice, the issue is more complex. The exposed population is extremely large in many endemic areas and testing for G6PD status in all individual is a costly exercise. Even testing in non-immune travellers returning from endemic areas is a daunting task given the numbers involved. Therefore, in many resource-limited settings, primaquine is prescribed without testing for G6PD status. The phenotypes of G6PD deficiency itself are highly variable, which is not surprising since there are almost 300 different allelic mutations for this X linked recessive disorder [1,29]. The mutations and prevalence of each mutation in a population determine the risk of 'blind' administration of primaquine. For example, G6PD deficiency is common among people of African origin but the enzyme deficiency is relatively mild (A^- ^variant) with most having greater than 10% of the enzyme activity [30]. They are relatively resistant to serious reactions with primaquine-induced haemolysis. However, among Caucasians and Asians, though the prevalence of G6PD deficiency is less, the enzymatic activity in homozygotes is relatively low leaving a sizable proportion of deficient individuals vulnerable to serious reactions [31]. The prevalence of G6PD deficiency also varies with the ethnic background. The Mediterranean B^- ^variant prevalent in certain ethnic groups originating in the Mediterranean and West Asia has minimal G6PD activity. They are vulnerable to severe life threatening haemolysis with primaquine [32].

Similar to sickle cell disease, the G6PD deficient states may also confer resistance against infection by *Plasmodium *sp. The rapid clearance and shorter half-life of red cells in affected individuals may be responsible for this effect. This phenomenon has been demonstrated for the African A^- ^variant, showing that it confers protection against lethal falciparum malaria [33,34].

Until recently, vivax malaria was not thought to confer such a benefit. Vivax malaria was considered a benign entity which could not exert a significant evolutionary selection pressure. However, recent studies have shown that in areas of vivax endemicity, G6PD deficiency might offer protection against the infection [35].

Leslie et al.[35] in a case control study of Afghan refugees showed that phenotypic G6PD deficiency was less common in cases with vivax malaria than in controls (1.1% vs. 5.7%). The adjusted odds ratio for hemizygous G6PD deficient males (Mediterranean variant) to contract vivax malaria was 0.12 (95% confidence interval 0.02-0.92, *p *= 0.041). Similarly, the adjusted odds ratio for females (heterozygous or homozygous) was 0.37 (95% confidence interval 0.15-0.94, *p *= 0.037). Another recent study has demonstrated that the G6PD Mahidol variant in Thailand is associated with a low rate of parasitaemia in infected adults in Thailand [36]. If this hypothesis of G6PD deficient states offering protection against vivax malaria is true, then it brings forth a dilemma. Assuming that most of the infected are unlikely to be G6PD deficient, prescribing primaquine may be relatively safe for radical cure. However, because of evolutionary selection, it might also indicate that the endemic areas have proportionately more G6PD deficient people, which might render non selective administration of primaquine in prophylactic regimens more dangerous. In some areas this proportion is estimated to be as high as 30% [37]. There is considerable variation between different ethnic groups, even if they are geographically close to each other. The estimated prevalence of G6PD deficiency in Central Asia varies from 2.1% in Tajikistan, 2.9% in Iran, 7.0% in Pakistani Pathans, 15.8% in Pakistani Pashtuns to as high as 36.4% in Azerbaijan [29,31,38,39]. Therefore, liberal prescription of primaquine cannot be carried out without assessing its risks in ethnic groups with high G6PD deficiency rates. Recommendations and precautions also have to vary according to the population prevalence of the condition.

In addition to the genotypic and phenotypic variations of the recipient, the dose of primaquine administered also influences the severity of the outcome in cases of G6PD deficiency. According to the current recommendations, patients may receive 0.75 mg base/kg body weight (45 mg stat dose) of primaquine for clearance of gametocytes of *P. falciparum *or 30 mg (or 15 mg/d as per WHO recommendation) daily doses for 14 days for radical cure of *P. vivax *[23]. In patients without G6PD deficiency, haemolysis is not observed even though primaquine is taken in daily maximum therapeutic doses for a prolonged period (up to 20 weeks in the trial by Baird et al. in Indonesia) [6]. In situations of G6PD deficiency, the primaquine dose and residual enzymatic activity will determine the severity of the manifestations of haemolysis. In mild deficiency states (African A^- ^variant) the haemolysis is mild and starts 3-4 days after exposure. The first to succumb are the aged red blood cells and the patients seem to tolerate the standard doses of primaquine after an initial episode of haemolysis. In fact, a safer approach in these patients would be to continue a weekly course of 45 mg of primaquine for 8 weeks and this has been demonstrated to be well tolerated without significant haemolysis [40-42]. More recent studies in Thailand and Burma also indicate that even in G6PD deficient individuals, massive haemolysis was not observed with standard doses of primaquine administration (45 mg stat doses and 15-30 mg daily for 14 days) [43-45]. However the numbers of G6PD deficient patients in all these studies were small and did not exceed 20. Nonetheless, these findings do not conclusively indicate that non-specific administration of primaquine is safe. If mass administration is allowed, millions of population will be exposed to the drug and the response of different genotypic variants of G6PD deficient individuals would be different. There may be sporadic but significant number of cases of severe haemolysis, haemoglobinuria, acute kidney injury and associated fatalities. This is especially true in certain geographical areas which have high G6PD deficient rates and in areas where the Mediterranean B^- ^variant is prevalent.

It is highly recommended to check for G6PD deficiency status in individuals before prescribing primaquine for terminal prophylaxis and in radical cure regimens. It is further recommended that governments in malaria endemic regions make an effort to quantify G6PD deficiency rates in different ethnic groups as this may be a long term investment in preventing primaquine induced adverse effects. Quantitative testing is expensive but now a qualitative fluorescent spot test is available which is sufficient to spot individuals with an increased risk [46]. Primaquine administration in pregnancy should remain contraindicated as the G6PD state of the foetus cannot be determined.

Methaemoglobinaemia is another consequence of primaquine administration which is commonly identified with therapeutic doses of primaquine; however, clinical effects are not usually seen (except possibly in individuals with gas diffusion defects in lungs and those with congenital NADH methaemoglobin reductase deficiency) [1,47]. Methaemoglobinaemia is defined as a level of more than 1% of the haemoglobin level [1]. Various studies have reported methaemoglobin levels up to 20% without any symptoms with therapeutic doses of primaquine [6,48,49]. Methaemoglobin levels also seem to be higher with higher doses of primaquine. The relationship between the duration of therapy and methaemoglobin levels is unclear. However, there must be other confounding factors for development of methaemoglobinaemia other than dose and duration, as not everyone shows a dose or time dependent rise of methaemoglobin levels following primaquine administration. In fact, this response is extremely variable. Again it may be genetically determined and there is increasing evidence that G6PD deficient individuals are more vulnerable to methaemoglobinaemia. However the evidence is not yet clear in this regard. Santana et al. [50] showed that in a group of patients developing methaemoglobinaemia following primaquine therapy, 51% were G6PD deficient (G6PD deficiency in those without methaemoglobinaemia was 8.7%). However, Ferreira et al. failed to demonstrate a similar association [51].

The other side effects of primaquine include gastrointestinal disturbances and hypersensitivity reactions (rare). In some, the gastrointestinal disturbances (nausea, vomiting) can be severe affecting compliance. It is recommended to take primaquine with food to avoid these unpleasant side effects. In all clinical trials on primaquine, it was well tolerated by most of the non-G6PD deficient patients or volunteers [6,11].

It is interesting to note a recent finding of primaquine induced differential gene expression in the liver with exposure to the drug in high doses in mice [52]. This study demonstrated that after being administered a single dose of primaquine at 40 mg/kg, there was a subsequent deregulation of RNA expression corresponding to several proteins involved in cellular processes such as intracellular signaling, protein transport, haemopoiesis, cell adhesion and proliferation. However, there were no biochemical alterations of hepatic enzyme levels in serum or microscopic histological abnormalities of liver sections. This is a preliminary animal study which needs to be explored further as consequences of long-term primaquine exposure in humans are yet unknown.

### Resistance to primaquine: Is it a major problem?

Primaquine has stood the test of time remarkably over six decades since its introduction in anti-malaria treatment. True treatment failure with primaquine is difficult to define due to the confounding factors mentioned below. However, it can be quoted as 'recurrence of parasitaemia following administration of a previously efficacious regimen for that region'. Resistance for primaquine mainly refers to its failure to clear the hepatic stages (hypnozoites) of the *P. vivax *life cycle, as it is not used as an erythrocytic schizontocidal agent.

Resistance to primaquine is a difficult entity to quantify due to following reasons. Firstly, primaquine is not used in isolation, it is combined with a blood schizontocidal agent, and the lack of efficacy between the two drugs is difficult to quantify separately [53]. Part of the efficacy of the standard chloroquine/primaquine regimen for confirmed cases of vivax malaria is due to the blood schizontocidal activity of the former.

Secondly, the duration of therapy is important, as primaquine is known to have a total dose effect on hypnozoites and hepatic schizonts of the parasite and shorter courses of primaquine have been largely proven to be ineffective. Therefore, failure reported in these instances does not reflect a true failure of primaquine.

Thirdly, there are many different daily dosing regimens that have been in use and the most popular was the 15 mg/d for 14 days regimen. Nonetheless, treatment success with this regimen was less than 100% in many trials [54-56]. The failure rates were less when the daily dose of primaquine was increased to 22.5 mg and 30 mg with the latter regimen having the highest efficacy [18]. In another trial, Pukrittayakamee et al. [57] report a higher efficacy in the treatment of acute *P. vivax *infections and prevention of recrudescence with a higher dose of primaquine (60 mg/d for 7 days compared to 30 mg/d), up to 28 days of observation.

Fourthly, addressing issues such as adequate dosing according to body weight and ensuring good compliance is necessary before announcing treatment failure [58]. A review of treatment failure with primaquine containing regimens in different endemic areas have shown that while adding primaquine to a blood schizontocidal agent considerably reduced the rate of relapse, the protective effect was more with a higher total dose of primaquine as well as a higher dose per body weight [59]. In some instances of reported failure with primaquine, inadequate dosing compared to body weight of individuals was obvious [22,59,60]. It also questions the validity of earlier case reports of treatment failure with primaquine containing regimens of 15 mg/d (or less than 0.5 mg/kg/d) over 14 days [61-66]. Non compliance is a serious problem with 14 day primaquine therapy. In many studies, the adherence was shown to be low even when a shorter course of primaquine was prescribed [67]. After the symptoms abate, patients may not complete the scheduled 14 days of treatment, especially because of gastrointestinal side effects. Takeuchi et al. demonstrated in a trial in the Thai- Myanmar border region, that with directly observed therapy (DOT), the compliance was better and the relapse rates were less compared to self-administered therapy [58]. Non-compliance with therapy was a major reason to push for a shorter course of therapy. Given the massive numbers involved, DOT may not be a feasible option in endemic areas like Africa and Southeast Asia. However, in countries on the eve of eradication of malaria with very low incidence rates, this may be a good option to execute via the public health monitoring system (similar to the surveillance and DOT of tuberculosis patients). For example in Sri Lanka, DOT for patients with malaria is already initiated. In other countries, the answer may lie with the potential alternatives to primaquine like tafenoquine which can be administered over a shorter duration (see below).

Fifthly, gauging the risk of relapse with primaquine failure is compounded by re-infection. The idea of radical cure with primaquine therapy is to remove hypnozoites that may cause treatment relapse. However, if the patient remains in malaria endemic areas, he/she might get re-infected with *P. vivax *again. This is not a treatment failure of primaquine. Yet in trials which assessed this aspect, there is little room to differentiate a relapse from a re-infection. There is no experimental methodology to assess the therapeutic efficacy of primaquine to clear the intra-hepatocyte stages of the parasite, although some researchers have made progress in this aspect in developing an *in vitro *assay [68]. Similarly, others have evaluated the possibility of including genotype analysis to differentiate between relapse and new infections in trials. Sanchez et al.[69] observed high rates of recurrences (up to 40% in 6 months) of *P. vivax *infections in two cohorts of patients treated with chloroquine and primaquine (30 mg/d for 7 days only) in the Brazilian Amazon. They performed genotyping in the *P. vivax *isolates of the initial infections and of the recurrent infections. A recrudescence would be characterized by the same genotype in both occasions and a new infection by a different genotype. However, a relapse (by activation of dormant forms) may be characterized by non-identical but related genotypes in the second infection. Of 28 pairs analysed, only two showed identical haplotypes and there were more non related haplotypes than related haplotypes favouring re-infection over recrudescence or relapse. However, interpretation and validation of such data is yet to be standardized. In a similar study where authors claim that the chances for re-infection were minimized, the genotypes of the acute and subsequent infections differed considerably [70]. This indicates three possibilities: a) several strains might have been inoculated in to the patient during the exposure period and each might have given rise to different episodes; b) simultaneous inoculations may occur at the time of initial bite by the mosquito; and c) high rates of mutations and genetic re-assortment of the hepatic schizonts may take place at the time of schizogony. Whatever explanation is the most likely, it casts serious doubts over the validity and usefulness of using genotyping to differentiate between a relapse and a re-infection.

Most of the reports of 'primaquine failure' are in relation to using it in radical cure regimens (as it is not routinely recommended as a causal prophylactic agent). A study by Park et al. gave a rare insight into its failure rates as a terminal prophylactic agent. They assessed the infection rate of vivax malaria in military personal being administered chloroquine throughout the exposure period, and primaquine as terminal prophylaxis. This was compared with the malaria incidence of soldiers not taking chemoprophylaxis. The rate of malaria did not differ significantly between the two groups. The estimated efficacy of primaquine as a terminal prophylactic agent was as low as 32%. However, the design of the study raises doubts about these figures. It is not clear when prophylaxis was administered and the time duration since prophylaxis to infection. This is crucial as there is no evidence as to how long the primaquine dose remains effective and when the person is vulnerable to re-infections. It is also not clear whether cases and controls had the same exposure to malaria for a valid comparison. If the controls did not receive chemoprophylaxis it is likely that their risk of exposure was less. The two groups having the same rate of infections may partly be due to new infections occurring after the chemoprophylactic effect had worn off. There was also no way to verify good compliance with the regimen as it was assessed using retrospective recall. More importantly, the chemoprophylactic regimen used was that of 15 mg/d for 14 days, which is currently considered inadequate for complete protection as per (CDC) recommendations.

A review by Baird et al. [71] of reported cases of primaquine resistance over three decades showed some interesting findings. A large number of reported treatment failures were with the shorter courses of therapy (15 mg/d for five days), which is considered ineffective. The number of cases of treatment failure with 15 mg/d for 14 days was considerably less. In most occasions of resistance, compliance could not be verified accurately. During the follow up period of 12 months, there was a progressive reduction in numbers of relapses as the daily primaquine dose increased (there were no relapses when 30 - 60 mg/d were administered for 14 and 7 days, respectively). However, even when a high dose of primaquine was administered without a concurrent schizontocidal agent, the relapse rates were heavy (up to 80%) [72,73].

Nonetheless, the laps and inadequacies of previously used primaquine regimens do not render primaquine resistance a non-entity. Though it is extremely difficult to establish true resistance, there are case reports of recurring vivax malaria despite an adequate dose of primaquine administered with an effective blood schizontocidal agent [19]. Many of these reports were from the Southeast Asian region and the resistance of the Chesson strain of *P. vivax *was an example of primaquine failure in the early days [74]. However, this was during the Second World War when there was mass migration of soldiers and populations and when many experimental drugs with a structural resemblance to primaquine were non-selectively distributed. In essence, the earlier reports of the Chesson strain of *P. vivax *indicate the potential for development of resistant strains when the environmental conditions and selection pressure favour it [1]. Even to date, failure with 15 mg/d for 14-day regimen is higher in travellers to Papua New Guinea (which was the focal point in which Chesson strain was isolated) than in other endemic areas [18].

Overall, a critical review of the reported instances of primaquine 'resistance' raises serious doubts over their validity with respect to design, adequacy of dosing, duration of treatment, risk of re-infection, non-compliance with unsupervised treatment and small numbers of patients in trials. It also indicates that success or failure of treatment cannot be defined for primaquine alone. It is more suitable to establish it with respect to the primaquine dose, duration and the concurrent blood schizontocidal agent administered, taken as a whole single entity.

### Primaquine with other blood schizontocidals against vivax malaria

Chloroquine has been the backbone of vivax treatment regimens since its inception and chloroquine together with primaquine was the standard therapy for vivax malaria on the understanding that *P. vivax *was universally sensitive to chloroquine. The first patient with chloroquine resistant vivax malaria was reported from Papua New Guinea in 1989 [75]. Since then cases have been reported from a vast geographical area spanning the continents of Asia (Indonesia, Myanmar, India, Turkey) [76-79], Africa (Ethiopia) [80] and South America (Brazil, Peru, Colombia) [81-83]. In response, several studies have evaluated the efficacy of primaquine with Artemisinin based Combined Therapy (ACT).

Krudsood et al. [84] assessed the efficacy of artemether-lumefantrine (each tablet containing 20 mg of artemether and 120 mg of lumefantrine, 24 tablets to be administered within 60 hours) vs. standard dose of chloroquine against vivax malaria in a randomized open labeled trial in Thailand. Both groups were given 15 mg/d primaquine for 14 days. Both regimens were equally effective in clearing the infection with only one treatment failure in the artemether-lumefantrine group. The authors concluded that it is an effective alternative to the current chloroquine based regimen. However, there was no follow up beyond 28 days to detect any relapses that would have assessed any impact on the concurrently administered primaquine. Similar findings were observed by Hamedi et al. [85] for artesunate and primaquine combination against vivax malaria. In a separate randomized trial, Krudsood et al. [86] assessed the efficacy of artesunate (600 mg daily for five days) and high dose primaquine (administered for 5 groups as 30 mg a day for 5, 7, 9, 11, and 14 days. Group 6 received primaquine, 30 mg twice a day for 7 days). The cure rates after 28 days of follow up were 100% and 96% for groups 5 and 6 respectively indicating the success of high dose primaquine with artesunate over a short observation period. The 60 mg/d dose was well tolerated and its short duration was thought to improve the compliance with the regimen, which is a major issue with the 14-day course. Dao et al. [87] in a smaller study (n-28) in Vietnam showed that artesunate (200 mg twice a day for 2 d) plus primaquine (22.5 mg base twice a day for 7 d) was highly efficacious in clearing the parasitaemia and ensuring cure at 28 days (only one treatment failure was observed). Another trial in Thailand compared four treatment arms; oral artesunate 5 day course (200 mg on D1 and 100 mg daily for remainder), artesunate 7 day course in a dose as above, artesunate 5 day course plus high dose primaquine (0.6 mg/kg/d × 14 days), artesunate 7 day course and high dose primaquine for 14 days [88]. The clinical response and parasitic clearance was rapid and similar in all four groups but almost 50% of participants who did not receive primaquine had recurrence of parasitaemia by D28. None of the patients who took primaquine had recurrent parasitaemia up to D28. Similar results were observed by Wilairatana et al. [20] who showed high dose primaquine and artesunate to be superior to sulphadoxine-pyrimethamine (SP), SP with high dose primaquine and high dose primaquine alone in clearing parasitaemia and avoiding recurrence up to 28 days.

Sinclair et al. [89] carried out a meta-analysis to assess the efficacy of ACT against uncomplicated vivax malaria. Of the 12 studies included in the analysis, only four had been given primaquine in an adequate dose to clear the hypnozoites. Even then, one trial had delayed the administration of primaquine till D28 and another had given an unsupervised primaquine course affecting the validity of evidence. It was concluded that artemisinins with a longer half-life such as dihydroartemisinin-piperaquine would provide a longer time of post treatment prophylaxis (up to six weeks) compared to artemether- lumefantrine even if concurrent primaquine was not administered. However, the role of primaquine with ACT cannot be elucidated from the trials mentioned in this meta-analysis.

Overall, it can be summarized that artemisinin-based therapy is a successful alternative to chloroquine based regimens in areas of vivax resistance. However artemisinins alone may not be enough to clear the hepatic schizonts and hypnozoites of *P. vivax *and, therefore, to achieve radical cure, primaquine in high doses must be added. The few trials available on these combinations (all from Southeast Asia) show that primaquine in high doses is safe, well tolerated and efficacious in preventing parasitaemia up to 28 days since initiation of therapy.

### Alternatives to primaquine: Can it be replaced?

Primaquine, despite being unique in its abilities, has several major disadvantages. As pointed out earlier, its potential to cause serious side effects of haemolysis in G6PD deficient individuals is the most important concern [1]. Its short half-life requires it to be administered over a course of 14 days which leads to poor compliance [58]. Circumventing this issue with shorter courses of therapy has been largely unsuccessful.

Seeking a replacement for primaquine did not gather momentum until recently due to several reasons. Many did not consider vivax malaria to be a serious illness till recent evidence proved otherwise (resistance to first line treatment, increasing incidence and severity of illness). The hunt for a replacement compound is also difficult when the mechanism of action of primaquine itself is obscure [18]. Furthermore, there is no proper *in vivo *or *vitro *assay process to determine the efficacy of primaquine as a hypnozoitocidal. There has not been any confirmed identification of intra-hepatocyte forms of *P. vivax *though some authors believe that they have been able to grow them *in vitro *cell cultures [68].

There are several alternative compounds that have been tested in various stages of clinical trials as a replacement for primaquine (tafenoquine, bulaquine, tinidazole and inidazolidinone) [90]. Bulaquine is the pro-drug of primaquine and is currently not licensed to be used outside India. Data on its safety with widespread use is not yet available [90]. Tafenoquine is a compound that has shown some promise when used in a shorter course of therapy of 3 days. It is also an 8-aminoquinoline as primaquine. Walsh et al. [56] in a randomized trial compared three different doses of tafenoquine (300 mg/d for 7 days, 600 mg/d for 3 days and 600 mg single dose) given one day after a blood schizontocidal treatment (chloroquine) in patients with vivax malaria. Additionally, these groups were compared with patients who received chloroquine alone and chloroquine with low dose primaquine (15 mg/d for 14 days). The protective efficacy of tafenoquine was significantly greater compared to other regimens up to eight weeks of follow up.

A larger, open-label, randomized, parallel-group study by Elmes et al. [91] in Australian defense force personnel returning from Timor-Leste and Bougainville (Papua New Guinea) have shown that tafenoquine containing regimens for 3 days caused less relapses than a primaquine regimen of 22.5 mg/d for 14 days when administered as terminal prophylaxis up to a follow up of 1 year. In addition to primaquine or tafenoquine, all received doxycycline as the primary prophylactic agent during the deployment. There were no significant differences of relapses between the primaquine treated and tafenoquine treated groups apart from the subset returning from Timor-Leste who received the lowest dose of tafenoquine (200 mg/d for 3 days) that had less relapses. Authors could not explain this discrepancy. However, it was noted that some time after the trial ended, the recommended dose of primaquine was raised to 30 mg/d and with that, the relapse rates were further reduced.

Kitchener et al. [92] assessed the efficacy of an extended course of tafenoquine in treating individuals with relapsing vivax malaria who were initially treated with primaquine (22.5 mg/d for 14 days) plus standard chloroquine treatment. Twenty-seven individuals (of Australian defense forces returning from deployment in the areas mentioned previously) were offered tafenoquine 200 mg base for 3 days and 200 mg weekly for 8 weeks subsequently. They were followed up for six months. There was only one relapse in this group, which already had treatment failure with primaquine at a dose of 22.5 mg daily. The tafenoquine-based long-term regimen had a cure rate of 96% in this study.

Tafenoquine is probably the most extensively studied compound showing hope of replacing primaquine in future. It has demonstrated efficacy at least comparable to primaquine in regimens of radical cure as well as in terminal prophylaxis. However, the pharmacodynamics of tafenoquine action on *P. vivax *is still not clear (e.g., the reason for a lower dose to be more effective than a higher dose in terminal prophylaxis). All the trials which compared tafenoquine against primaquine had done so for a lower dose of primaquine than what is recommended now. There may not be any advantage of tafenoquine had they compared it with a 30 mg/d course of primaquine. The issue of haemolysis with G6PD deficient individuals still remains a risk with tafenoquine. All participants were cleared as non-G6PD deficient prior to enrolment in the trials and the safety of tafenoquine against haemolysis is unknown. Probably the only potential advantage tafenoquine carries is its ability to ensure a better compliance with therapy as the treatment course can be shortened to three days.

## Conclusions

The conclusions of this review as per current evidence on primaquine are as follows;

a) Primaquine is efficacious for its current suggested indications in relation to vivax malaria, namely; causal prophylaxis (in special circumstances), terminal prophylaxis, and radical cure. It is the only licensed drug in use against the intra-hepatic forms (schizonts and hypnozoites) of *P. vivax *but its efficacy is highly dependent on the concurrent administration of a blood schizontocidal agent. The most established drug in this regard is chloroquine. The currently recommended dose against vivax malaria is 0.5 mg/kg/d not exceeding 30 mg/d for 14 days for radical cure according to the CDC guidelines. However, WHO still recommends a lower dose except for Oceania and Southeast Asia. We feel that the evidence supports the use of the higher dose in preventing treatment failure in other regions as well.

b) G6PD deficiency state can cause severe haemolysis with primaquine and it is a barrier against the widespread use of the drug. Since its prevalence and severity varies with geographical location as well as ethnic origin of populations, it is difficult to propose universal guidelines in this regard. It is recommended that local governments in endemic areas make a conscious effort to quantify the prevalence of G6PD deficiency as it will have a major effect on the control of vivax malaria. Qualitative assessment with fluorescent testing may be cost effective in this regard. Other side effects, such as methaemoglobinaemia and gastrointestinal side effects, did not pose a serious threat to health in clinical studies.

c) Resistance to primaquine is a doubtful entity. Most cases of so-called 'resistance' might have been averted with a higher dose of primaquine. The validity of reports is contaminated by errors in trial design, risk of re-infection, inadequate dosing and unsupervised treatment. Furthermore, primaquine does not act in isolation and resistance to it (if any) has to be defined in relation to the concurrently administered blood schizontocidal agent as well.

d) Artemisinin based therapies are a successful alternative in areas of chloroquine resistance against vivax malaria. However primaquine in high doses has to be administered concurrently to avert relapses. A few trials in Southeast Asian region show that primaquine in high doses is safe, well tolerated and efficacious in preventing parasitaemia up to 28 days since initiation of therapy in combination with artemisinins.

e) Tafenoquine is a drug with promise as an alternative to primaquine. However, its only advantage over primaquine may be the shorter duration of treatment. Neither its effect in relation to G6PD deficiency, nor its efficacy compared with currently recommended treatment, has been established. Further studies are needed in this regard.

## Abbreviations

Abbreviations are explained where they are first used in text.

## Competing interests

The authors declare that they have no competing interests.

## Authors' contributions

All authors have participated in designing, article search, information coding and writing of the manuscript. All authors have read and approved the final manuscript.
